# Towards a unifying theory of linguistic meaning

**DOI:** 10.1080/19420889.2023.2200666

**Published:** 2023-04-26

**Authors:** Prakash Mondal

**Affiliations:** Department of Liberal Arts, Indian Institute of Technology Hyderabad, Kandi, Sangareddy, India

**Keywords:** formal-logical structures, cognitive representations, linguistic meaning, brain dynamics, neurobiology, unifying theory

## Abstract

Fundamental tensions exist between formal-logical approaches and cognitive approaches to linguistic meaning. The divergence arises from the fundamental differences in nature and form between formal/mathematical structures of natural language meaning and their cognitive representations. While the former are abstract and logical categories of representations, the latter are ultimately embodied and grounded in sensory-motor systems of the brain. This article aims to motivate a unifying theory/formalism of linguistic meaning from a general biologically integrative perspective in the context of current theorizing in linguistics, neurobiology and cognitive sciences on human language meaning within which two divergent approaches for the mathematical and cognitive aspects of linguistic meaning exist. The tensions can be somewhat neutralized if formal-mathematical structures and cognitive representations of natural language meaning can be shown to have representational duality and unity in brain dynamics. This work shows a broad outline of one, if not the only one, path toward this vision.

## Introduction

Formal-logical approaches and cognitive approaches to linguistic meaning have for decades remained in conflict. The conflict can be partly traced to the thinking of Frege [1] who did not consider psychological states to figure in the description of natural language meaning. Even though Frege [2] was cognizant of the import of both cognitive and logical facets for the description of linguistic meaning, the logical facets of linguistic meaning and the kind of inferences they admitted of were of central concern to Frege. This became consolidated with the appearance of Montague’s intensional logic that offered a way of translation of linguistic meaning in logical formulae which were functions from worlds and times to truth values [3] p. 228). In contrast, cognitive approaches to linguistic meaning [4–7] emerged against the background of the cognitive revolution in the 1950s and, in part, as a reaction to the Chomskyan theory of syntax that divorced semantics from syntax. Thus, embodied cognitive representations and formal/mathematical structures of linguistic meaning appear to be irreconcilable aspects of natural language meaning. It is time to seek a unifying theory of linguistic meaning that can show that cognitive representations and formal-logical structures of linguistic meaning are one and the same thing.

## An overview of the tension

**Figure 1.**
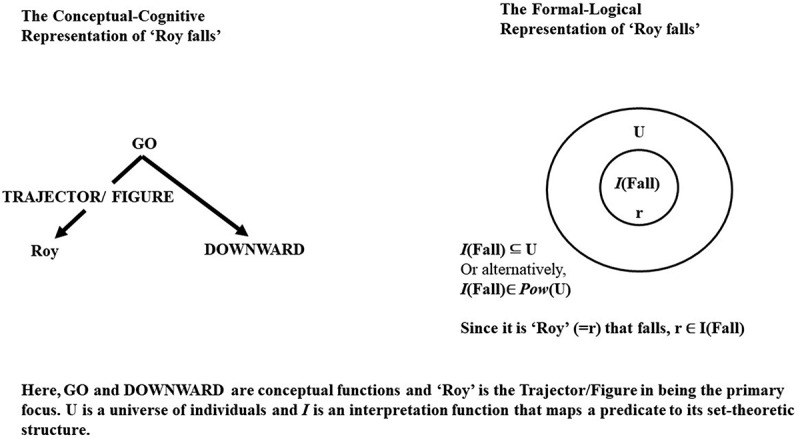
The conceptual-cognitive representation of ‘Roy falls’ juxtaposed with the formal-logical representation.

Natural language meaning can be characterized in terms of not only cognitive representations but also formal-logical structures. On the one hand, cognitive representations of natural language meaning go well with embodied theories of cognitive representations that trace mental representations and structures to our sensory-motor systems interfacing with the outer world [8]. One important clarification is in order here. Although cognitive representations of natural language meaning dovetail with sensory-motor embodiment, not all theories of cognitive representation *in general* embrace embodied views of cognition, since most scholars in classical cognitive science, motivated by the computer metaphor of the mind, think that cognitive representations are devoid of sensory qualities and contents, and also that they are more like sentences [9] see for discussion [10]. On the other hand, formal-logical structures are least embodied in sensory-motor systems, precisely because they are abstract categories and, hence, they are not aspects of brain structure [11]. For one thing, formal-logical patterns are valid not because they are patterns of brain structure but because formal-logical patterns are necessary in terms of symbolic rules and so valid, and hence our brains come to encode logical patterns [12]. For another, formal-logical patterns are patterns and categories of the outer world, even though they can be mentally constructed by humans and imposed on the outer world in conformity with certain observed regularities. The composition of linguistic meaning in this tradition is logical and program-like in being solely concerned with set-theoretic structures and truth values [13]. Meaning is thus represented as a formal object^1^ that is compositionally derived from (parts of) linguistic expressions in formulas of logics but the most striking point is that the composition of linguistic meaning has no psychological basis. Even if the composition of linguistic meaning is conceived in psychological terms, the composition functions or operations, as mathematically defined, are not usually thought to be proof-theoretic procedures in the mind, and since the objects to be combined are concepts or cognitive representations rather than set-theoretic entities, it is not clear how concepts or cognitive representations can be put together in just the way proof-theoretic or model-theoretic composition functions demand (see [15,14]. Other formal models of linguistic meaning such as vector space models [16] or semantic networks [17] have also been formulated along somewhat similar lines but they are more attuned to words and their commitment to the compositionality of linguistic meanings is somewhat weak. In contrast, with the emergence of cognitive approaches to linguistic meaning, linguistic meaning has come to be understood in terms of representations, structures and patterns in the mind [4–7]. Thus, linguistic meaning on this approach is constituted by mental representations and structures ultimately rooted in sensory-motor systems. Clearly, tensions exist between the formal-logical properties of linguistic meanings, which are abstract and seem to be properties of a non-biological realm, and the cognitive properties of linguistic meanings, which are patterns of structures to be found in brains/minds. For instance, the verb ‘fall’ is conceptually decomposed in terms of the motion-based conceptual function GO and the path DOWNWARD in Jackendoff [4]p. 366), where the agent argument of GO will be Langacker’s Trajector [5]p. 231 [6]; p. 8) or Talmy’s Figure [7] p. 311). In contrast, ‘fall’ is characterized in standard formal-semantic terms as a set of individuals/entities that fall. If our example sentence is ‘Roy falls’, Roy is supposed to be a member of the set of individuals that fall and also of the domain of all individuals (say, a universe) in a given context. It is necessary in virtue of the symbolic rules of logic that if Roy is a member of the set of individuals that fall and also of the domain of all individuals, Roy is also a member of the intersection of the set of individuals that fall and the domain set of all individuals. All this is schematized out in Figure 1. Clearly, these logical patterns/properties do not simply arise from the conceptual/cognitive representations of ‘fall’ in terms of a combination of motion and path.

The fundamental tensions derive from the *internalist* nature of conceptual/cognitive representations and *externalist* orientation of formal-logical structures. The common understanding is that such internalist conceptual/cognitive representations are in some sort of *language of thought* in Fodor’s [18] sense (see [19] p. 47). However, it may be noted that Fodor [20] p. 98–99) does subscribe to an externalist version of semantic structures, Chomsky [21] does not support the externalist orientation of semantic representations, although Chomsky does not conceive of semantic structures along the lines formulated in Cognitive Semantics by Langacker and Talmy, or in Conceptual Semantics by Jackendoff. Interestingly, like Chomsky, Jackendoff [4] thinks that conceptual structures of linguistic meaning are not representations of anything in the world and hence do not mean anything; rather, they are meanings. Regardless of whether or not the internal cognitive representations of linguistic meaning can have an externalist orientation, it is clear that cognitive representations can have a combinatorial structure^2^ at a descriptively functional level, as in Fodor’s language of thought, but are ultimately rooted in sensory-motor systems of the brain, unlike the language of thought. This is exactly what makes cognitive representations of linguistic meaning interface with the outer world. This way symbolic conceptual/cognitive representations can facilitate the operation of cognitive tasks in perception, reasoning, planning, etc. in relation to things in the outside world (see [22].

We may now wonder if there is empirical support for either type of approaches to linguistic meaning. This is what we turn to now. On the front of cognitive approaches to linguistic meaning, there is neuroscientific evidence that suggests that parts of the brain are differentially activated for different components of cognitive representations of verbs [23,24], p. 152–153). At the same time, it is also empirically established in embodied cognitive science that linguistic meanings are grounded in sensory-motor systems [25]. On the other hand, it is becoming evident that the acquisition of formal-logical properties of linguistic meaning by children supports the formal basis of linguistic meaning [26]. Besides, the emerging neuroscientific evidence reveals that cognitive representations and formal-logical, referential properties of linguistic meaning are co-activated and part of parallel, shared streams of processing in the brain (see [27,28].

On the other hand, in the face of tensions between cognitive representations and formal-logical properties of linguistic meaning, theoretical linguists, especially semanticists, and cognitive scientists have complained about the fundamental lacuna in the understanding of what linguistic meaning is [29,30]. The need for integration of formal-logical properties of words with conceptual representations has been felt [31]. Since linguistic meaning is notoriously hard to define or measure using standard quantitative methods, it is really hard to understand how linguistic meaning can come to have both abstract/logical structures and cognitive representations that can be measured or detected in experimental studies. Existing approaches toward the neutralization of the tensions between formal and cognitive approaches to linguistic meaning have pointed to the possibility of there being some sort of compatibility between formal-logical characterization of linguistic meaning and cognitive representations [32]. The idea is simply that cognitive representations can be assigned set-theoretic interpretations. Formulating a single ensemble of combinatorial processes for both human language and conceptual processes can form the basis of the said compatibility. Needless to say, a demonstration of compatibility is not tantamount to unification. The philosopher Philip Kitcher [33] has argued that unification in science, as in Maxwell’s unification of electricity and magnetism, or Darwin’s unification of life forms, can proceed when the same stringent pattern can help derive descriptions of many apparently divergent phenomena. Therefore, in the present case what is desired is a unifying formalism/theory that can show not only that formal-logical structures and cognitive representations of linguistic meaning are one and the same thing but also that they are mathematically equivalent. The first one is an ontological puzzle – a harder one, whereas the second one can be established with some degree of formal sophistication.

So, the unification must proceed along two parallel lines: one is ontological and the other one is formal and/or theoretical. On the one hand, ontological unification may look like a kind of reduction of formal-logical structures to cognitive representations of linguistic meaning, and vice versa, but it need not be so. It can be a matter of integration of two kinds of description of different ontological depths. On the other hand, the mathematical unification may be a matter of inter-translation and logical explanation/derivation of formal-logical structures in terms of cognitive representations of linguistic meaning and vice versa.

## Toward a unified theory

It is crucial to recognize that a unified theoretical account of formal/mathematical structures and cognitive representations of linguistic meanings cannot be merely a computational model of embodied linguistic meanings encoded in terms of set-theoretic and/or other similar mathematical structures (say, vectors and matrices). The reason is that many cognitive aspects of linguistic meaning can be described by mathematical structures and a unified theoretical account has to say something over and above a mere mathematical description of linguistic meaning. What is instead required is that the theoretical unification should show embodied cognitive representations to be identified and integrated with formal-logical properties of linguistic meanings and vice versa [11]. While it is clear that the chasm between cognitive representations and formal-logical properties of linguistic meanings exists and needs to be bridged, the present work specifically outlines the explanatory and methodological scope of a unified theory that can potentially eliminate the chasm. In any case, the general formulations and principles must be such that formal-logical properties of linguistic meanings and embodied cognitive representations can be shown to be equivalent. This will serve to establish the representational or formal equivalence between formal-logical structures and embodied cognitive representations of linguistic meaning.

It is now worthwhile to describe the kind of experimental work whose findings can potentially support the unified formalism of linguistic meaning. Such experimental findings in semantics and/or cognitive science will be exactly the ones in which the contributions of cognitive representations and formal-logical structures to linguistic meaning can be clearly spelt out (see, for instance [34]. If the contributions of either cognitive representations or formal-logical structures cannot solely account for the findings, then unambiguous evidence for the unified formalism/theory will accrue. There is psychological evidence from the study of cognitive dynamics of negation in Dale & Duran [35] that shows that non-facilitative contexts of negation processing trigger discrete shifts in responses recorded through computer mouse trajectories, whereas facilitative contexts of negation processing with enriched contexts facilitated the construction of mental spaces of negated meanings. Findings like these cannot be accounted for with either cognitive approaches or formal-logical approaches alone.

In addition, despite the ontological divergence between cognitive representations and formal-logical structures of linguistic meaning, brain dynamics (time-dependent state changes of the brain modeled as a multi-dimensional system, as in dynamical systems theory) can exhibit the *emergent* manifestation of convergence between cognitive representations and formal-logical structures of linguistic meaning. Brain dynamics rather than spiking activities in populations of neurons or neural activations in fixed locations will fit the bill for the convergence between cognitive representations and formal-logical structures of linguistic meaning. That is because patterns of linguistic meaning for linguistic expressions are higher-level constructions to be found at a scale/level higher than the one for neuronal spikes or localized activations. Fortunately, a dynamical account of the relation between symbolic dynamics and neurodynamics can prove helpful [36]. The underlying idea is that cognitive representations and formal-logical structures of linguistic meaning are higher-level states in brain dynamics. The higher-level states (state-space changes in brain dynamics) ride on the lower-level states of brain dynamics (flow of neuronal activations in neurons and neuronal assemblies). Such higher-level states (that is, macrostates) are *contextually emergent* over the lower-level ones (that is, microstates), exactly when the lower-level description is necessary but not sufficient for the higher-level description [37] 179). Thus, by following Maimon & Hemmo [38], we may say that a macrostate in brain dynamics is the complete description of the physical state of neuronal activations in neurons and neuronal assemblies at a given time, whereas a partial description of the set of all microstates across time is a macrostate. This can be described in other words. The dynamical system of the brain with a state space (the set of all possible states) can be in a specific state in terms of a description, especially when the system is in one of a number of other states that are part of another description. The former description defines a microstate and the latter defines a macrostate, since the latter is a kind of *coarse-grained* description or a partition of the former.

Despite the fact that formal-logical structures of linguistic meaning are abstract categories, they can ride on conceptual-cognitive representations of linguistic meaning in order to be part of actual semantic interpretations. That is because formal-logical structures of linguistic meaning have to be learnt by children anyway. Children extract formal-logical generalizations about verbs and constructions on the basis of conceptual-cognitive representations of specific verbs or verb classes along with their argument types [39,40]. This suggests that even abstract categories like formal-logical structures of linguistic meaning have to align with conceptual-cognitive representations of linguistic meaning. Besides, even abstract categories such as membership functions can be identified with coherent patterns in neural networks that can be interpreted as cognitive representations [41]. In a nutshell, the underlying message is that even if formal-logical structures of linguistic meaning are not *intrinsic* aspects of brain structures, they are processed, acquired or learnt by human brains. Therefore, they must have to coincide with cognitive representations of linguistic meaning in some way. On the linguistic front, what this means in concrete terms is simply that formal-logical structures (set-theoretic interpretations) have to be assigned to words so that their composition can give rise to complex meanings, and this will have a corresponding organization at the level of cognitive structures, schemas, or representations [42]p. 70–71). The import of it all can be appraised with the help of the following correspondences between formal-logical structures and cognitive representations for proper names, quantified expressions, coordinate structures, negation, etc., of course, constrained by the general formulations in Mondal [11]p. 7–8).

(1) c ≡T/τ

Here, a proper name (such as ‘Roy’, ‘Joy’, etc.) is designated as a constant (c) on the left-hand side of (1) in formal-semantic terms, whereas in cognitive representation on the right-hand side of (1) it would simply be an object type (T) with only one instance (τ), unless a proper name is used like a common noun, as in ‘There are two Roys in this class’. (see [43] p. 316–318). So, since any name is part of a network of extended knowledge structures, if any stereotypical features associated with the name change, we may still recognize the person or thing referred to by the name, mainly because the name is part of a stable mental model. Now we turn to quantified expressions. If we have sentences such as ‘A cat is in the room’, ‘Two cats are in the room’, ‘Cats are lovely’, ‘Most cats are in the room’, etc., correspondences need to be drawn between their formal-logical structures and the cognitive representations. Jackendoff’s conceptual functions come to be handy in serving the purpose of combining cognitive representations.

(2) $$\;\;\;\;\;\;\;\;\;\;\;\;\;\;\;\;\;\;\;\;\;\;\;\;\;\;\;\;\;\;\;\;\;\;\;\;\;\;\;\;\;\;\;\;\;\;\;\;\;\;\;\;\;\;\;\;\;\;\;\;\;\;\;\;\;\;\;\;\;\;\;\;\;\;\;\;\;\;\;\;\;\;\;\;\;\;\;\;\;\;\;\;\;\;\;\;\;\;\;\;\;\;\;\;\;\;\;\;\;\;\;\;\exists x\left[Cat\left(x\right)\wedge In\left(x,r\right)\right]\equiv \exists T^{R}:cat\;BE\left({\;}_{Object}^{\;}T^{R}\left[+INDEF\right]{,}_{Place}IN\left({\;}_{Object}^{\;}Room\left[+DEF\right]\right)\right)$$

By following Langacker [6]p. 255–257) and Jackendoff [4]p. 406), the cognitive representations have been schematized on the right-hand side. Here, r=Roy and ‘Cat’ and ‘In’ are predicates on the left-hand side, and on the right-hand side, T^R^ is the Trajector of the situation described and it indicates an arbitrary *representation-instance* of a reference quantity (R), thereby reflecting the intuition that ‘a’ as an indefinite determiner/existential quantifier represents an arbitrary instance from a quantity of a certain class. BE and IN are conceptual functions as specified in Jackendoff [4]p. 364)—BE takes two conceptual objects, namely, T^R^ and a place. A place is further specified by IN which takes an object, namely, ‘room’. Now, (3) specifies the desired correspondence for the sentence ‘Two cats are in the room’.

(3) $$\begin{array}{rl} & \exists x\exists y\left[Cat(x)\wedge Cat(y)\wedge In(x,r)\wedge In(y,r)\right]\equiv \exists ({T_{1}}^{R},{T_{2}}^{R})\\ & :cats:T^{R}\;BE{(}_{object}T^{R}\left[+PLURAL\right]{,}_{place}IN{(}_{object}Room[+DEF])) \end{array}$$

Now, we can have (4) for ‘Cats are lovely’. On the left-hand side below, X is a higher-order variable ranging over a set of entities, and Cat* is a higher-order predicate taking a set as an argument, and **T**^R^ on the right-hand side specifies a generic type of instances, admitting of a universal construal such that the proportion selected out of R equals R [6] p. 253–254).

(4) $$\;\;\;\;\;\;\;\;\;\;\;\;\;\;\;\;\;\;\;\;\;\;\;\;\;\;\;\;\;\;\;\;\;\;\;\;\;\;\;\;\;\;\;\;\;\;\;\;\;\;\;\;\;\;\;\;\;\;\;\;\;\;\;\;\;\;\;\;\;\;\exists X\left[Cat^{*}(X)\wedge Lovely(X)\right]\;\equiv \exists T^{R}:cats\;BE\left({\;}_{object}T^{R}\left[+PLURAL\right],{\;}_{Property}Lovely\right)$$

Finally, (5) captures the correspondence between the formal-logical structure and the cognitive representation of linguistic meaning for ‘Most cats are in the room’.

(5) $$\;\;\;\;\;\;\;\;\;\;\;\;\;\;\;\;\;\;\;\;\;\;\;\;\;\;\;\;\;\;\;\;\;\;\;\;\;\;\;\;\;\;\;\;\;\;\;\;\;\;\;\;\;\;\;\;\;\;\;\;\;\;\;\;\;\;\;\;\;\;\;\;\;\;\;\;\;\;\;\;\;\;\;\;\;\;\;\;\;\;\;\;\;\;\;\;\;\;\;\;\;\;\;\;\left(most\;x:Cat\left(x\right)\right)\;In\left(x,r\right)\equiv most\;T^{R}:cats^{.}BE\left({\;}_{Object}^{\;}T^{R}\left[+PLURAL\right],{\;}_{Place}IN\left({\;}_{Object}^{\;}Room\left[+DEF\right]\right)\right)$$

As far as coordinate structures and negation are concerned, correspondences can be drawn up for them along similar lines. If we have a sentence such as ‘Roy is smart and Joy is not weird’, both coordination and negation can be specified as part of the desired correspondence between the formal-logical structure and the cognitive representation. So, we can have (6) where AND is the operator of conjunction and NEG is the negation operator.

(6) $$\;\;\;\;\;\;\;\;\;\;\;\;\;\;\;\;\;\;\;\;\;\;\;\;\;\;\;\;\;\;\;\;\;\;\;\;\;\;\;\;\;\;\;\;\;\;\;\;\;\;\;\;\;\;\;\;\;\;\;\;\;\;\;\;\;\;\;\;\;\;\;\;\;\;\;\;\;\;\;\;\;\;\;\;\;\;\;\;\;\;\;\;\;\;\;\;\;\;\;\;\;\;\;\;\;\;\;\;\;\;\;Smart\left(r\right)\wedge \neg Weird\left(j\right)\equiv BE\left({\;}_{Object}^{\;}Roy,{\;}_{Property}Smart\right)AND\;BE\left({\;}_{Object}^{\;}Joy,{\;}_{Property}NEG\left(Weird\right)\right)$$

This exercise is meant to show that both formal-logical structures and cognitive representations of linguistic meaning can be higher-level patterns in the brain. All this indicates that cognitive representations and formal-logical structures of linguistic meaning can be interpreted as macrostates in brain dynamics. Both cognitive representations and formal-logical structures of linguistic meaning in being macrostates should meet the condition of being *topologically equivalent* to the underlying neuronal dynamics, that is, microstate dynamics. In other words, both cognitive representations and formal-logical structures of linguistic meaning as higher-level states in brain dynamics should be structurally invariant with respect to the underlying neurodynamics. This indicates that even though cognitive representations and formal-logical structures of linguistic meaning appear different in representation and form, brain dynamics should display the relevant convergence between them. Now, this also implies that if cognitive representations and formal-logical structures of linguistic meaning are *one and the same thing* when they converge in brain dynamics. The underlying brain dynamics may evince this in terms of the right **microstate-to-macrostate mapping** (for the equivalence between cognitive and formal/logical representations), **uniformity** (for the stability in higher-level brain dynamics for cognitive and formal/logical representations, despite variation in neuronal dynamics at lower/micro-level scales) and **congruency** (overlapping or identical dynamical behavior for translations/encodings of cognitive representations into formal/logical representations and vice versa) in symbolic dynamics between cognitive representations and formal-logical structures of linguistic meaning [11]. Given the current research on the viability of vector-based representations of linguistic meaning in neuronal dynamics of semantic composition [44], the conditions of mapping, uniformity and congruency vis-à-vis lower-levels of neuronal dynamics in specific brain regions can be put to the test. At this point, two important predictions can be offered.

**Prediction 1**. The typical characteristics and properties of cognitive representations of linguistic meaning (as described and illustrated in the second section) will be manifested in formal-logical structures of linguistic meaning in semantic interpretation and reasoning, and/or vice versa. In other words, even if formal-logical patterns of linguistic meaning are confirmed to be found in semantic interpretation and reasoning, they cannot be fully explained with formal-logical patterns alone. Or conversely, even if cognitive representations of linguistic meaning are found in semantic interpretation and reasoning, they cannot be fully explained with patterns of cognitive representations alone.

**Prediction 2**. If cognitive representations and formal-logical structures of linguistic meaning converge in brain dynamics, and *yet* they appear different in representation and form, then the conditions under which they tend to appear different must be different too. Certain pressures and constraints from sensory-motor, memory and affective systems in the brain may induce the manifestation of cognitive representations on the one hand, and, on the other hand, gradual abstraction in a formal-logical sense may induce the emergence of formal-logical structures of linguistic meaning in meaning interpretation and reasoning.

New experimental studies can be conducted to put these predictions to the test (the outline of a sample experimental design has been provided in the Appendix as an illustrative case) and existing experimental findings can be checked to see if they are explained in a more adequate manner with the unified formalism/theory. The experimental studies that can test the predictions above will contain, as part of the experimental designs, precisely articulated contributions of cognitive representations and formal-logical structures of linguistic meaning such that the findings cannot be explained with just one kind of representations/structures. Some studies such as Dale & Duran [35] buttress Prediction 2. Besides, there are others that support Prediction 1. For instance, Smirnova & Lliev [45] and Smirnova [46] have conducted experimental studies that have investigated the formal-logical and cognitive differences between evidential (such as ‘It may/must have rained’) and non-evidential sentences (such as ‘It rained’). What these studies have found is that the participants judge evidential sentences to be weakly true (in terms of a scale), which coincides with less certainty and more cognitive distance from the propositions judged to be under the scope of an evidential, whereas non-evidential sentences are often judged to be (strongly) true. Interestingly, as the findings in these studies show, the tendency to consider evidential sentences to be less likely to be true harmonizes well with the observation of the same participants’ cognitive distance from the propositions under an evidential. Since cognitive representations of evidential sentences consist in some sort of cognitive distance between an agent’s conception of reality and the proposition that is evaluated (see for general discussion [4], 402–406), this nicely dovetails with a modal construal of evidential sentences [47] p. 17) and remarkably brings both formal-logical (truth-valued) judgments and cognitive representations into manifestation over one another (in line with Prediction 1).

This way the unified formalism can prove to be theoretically and conceptually expansive in offering the desired synthesis and also in having far wider explanatory coverage than purely conceptual/cognitive theories of linguistic meaning or purely formal-logical approaches. The hope is that further experimental findings of the sort that can test the predictions articulated above can further corroborate the unified theory of linguistic meaning.

## Implications and consequences

The unification of cognitive representations and formal-logical structures of linguistic meaning can have expansive and wide-ranging consequences for linguistic and cognitive sciences. Theories of semantic representation will be greatly simplified with the unified formalism/theory of linguistic meanings. The typological description of semantic structures across languages can also benefit from the unified representation of linguistic meanings, in that the unified structure will afford wider descriptive and explanatory efficacy in helping ferret out diverse patterns of linguistic meaning across languages. A deeper understanding of the brain representation of linguistic meanings can be achieved with the help of a unified formalism/theory of linguistic meaning. Also, the implementation of linguistic meanings in AI systems will be seamless as the knowledge representation of semantic structures can be more uniform than ever. This is so because the mathematical structure of the equivalence between cognitive representations and formal-logical structures of linguistic meaning (see for detail [11], will offer a single system of representation for linguistic meaning. As the study of linguistic meaning in theoretical linguistics can be integrated with neuro-cognitive/experimental semantics, computational-neuroscientific studies of neuronal networks, and AI research in knowledge representation of linguistic meaning, we can hope for a confluence of insights across linguistic and cognitive sciences. All these will ultimately help integrate the cognitive sciences in a much more inclusive manner – something that is presently missing in current linguistic and cognitive sciences.

## Notes

[1] It needs to be clarified that Chomsky’s [48] Minimalist syntax takes syntactic expressions to be formal objects, but this does not mean that formal semantics and Minimalist syntax are on the same formal footing. While Minimalist syntax takes syntax to be a formal system grounded in the mind/brain, formal semantics deals with formal-logical patterns of meaning that have an externalist orientation. This paper refers to the latter, when talking about formal-logical structures of linguistic meaning. Besides, Chomsky’s [48] Minimalist syntax does not theorize about linguistic meaning, except for making room for logical representations that are constrained by syntax (also called Logical Form).

[2] Cognitive representations in Fodor’s *language of thought* have a combinatorial syntax akin to that of natural language such that if A is a thought and B is also a thought, then A and B can be combined, for example, to form a complex thought A ˄ B.

## Appendix

In outline, a representative experimental design of the sort required to test Prediction 1 and Prediction 2 may involve checking if sentences under certain constructional types are interpreted in ways that lend credence to either formal-logical structures or cognitive representations, or to both, in reasoning. Such constructions need to be carefully chosen, primarily because components of formal-logical structures and those of cognitive representations need to be precisely specified. So, some illustrative cases can include causative constructions (such as ‘Joy melted the butter’), change-of-state constructions (such as ‘Joy opened the door’), and motion-path constructions (such as ‘Joy ran into the house’). The test sentences can be presented to the participants of the study in two phases—one phase will involve the presentation of sentences to be (randomly) evaluated for either truth-value judgments or some specific construal of cognitive representations, and the other one will involve the presentation of sentences in a mixed condition, consisting of response choices favouring truth-value judgments and the construal of cognitive representations. Some examples of the former are provided below—(i-iii) can test for formal-logical structures and (i’-iii’) can test for cognitive representations of meaning.

(i) Joy melted the butter and it is not Joy who did it.

**Options**: A: (i) is consistent, B: (i) is inconsistent.

[checking if j=Joy is part of the ordered pair <j, b> ∈ I(melt)]

(ii) Joy opened the door and it is not the door that Joy opened.

**Options**: A: (ii) is consistent, B: (ii) is inconsistent.

[checking if b=the butter is part of the ordered pair <j, b> ∈ I(melt)]

(iii) Joy ran into the house and it is not running that Joy did.

**Options**: A: (iii) is consistent, B: (iii) is inconsistent.

[checking if the predicate is ‘melt’)]

(i’) Joy melted the butter, which means that …

**Options**: A: the butter is melted, B: the butter is not melted.

[checking the result state component of (i)]

(ii’) Joy opened the door, which means that …

**Options**: A: the door did not open on its own, B: the door opened on its own.

[checking the causation component of (i)]

(iii’) Joy ran into the house, which means that …

**Options**: A: Joy moved from point X (outside the house) to point Y (inside the house), B: Joy moved from point Y to point X. [checking the path component of (i)]

In contrast, the mixed condition will involve a mixture of options from both groups above. Since this is a commentary paper, conducting a study of this type is beyond the scope of this paper. This may be left open to future investigations along this line.

## References

[1] Frege G (1892). Über sinn und bedeutung zeitschrift für philosophische kritik, 100, 25–30. In M. Black & P. Geach (Eds.), (1952). *Translations from the philosophical writings of Gottlob Frege* (pp. 56–9). Oxford: Blackwell.

[2] Frege G. Posthumous writings. P. Long & R. White, Translated by. Oxford: Blackwell; 1979.

[3] Montague R. The proper treatment of quantification in ordinary English. In: Hintikka KJJ, Moravcsik JME Suppes P, editors. Approaches to natural language. Dordrecht: Reidel; 1973. pp. 221–242.

[4] Jackendoff R. Foundations of language: brain, meaning, grammar, evolution. New York: Oxford University Press; 2002. 10.1017/s0140525x03000153 15377127

[5] Langacker R. Foundations of cognitive grammar. Stanford: Stanford University Press; 1987.

[6] Langacker R. Grammar and conceptualization. Berlin: Mouton de Gruyter; 1999.

[7] Talmy L. Toward a cognitive semantics: concept structuring systems. Vol. 1. Cambridge, Mass: MIT Press; 2000.

[8] Shapiro L. Embodied cognition. London: Routledge; 2019.

[9] Fodor JA, Pylyshyn ZW. Connectionism and cognitive architecture: a critical analysis. Cognition. 1988;28:3–71. 2450716 10.1016/0010-0277(88)90031-5

[10] Fresco N. Physical computation and cognitive science. Berlin: Springer Nature; 2014.

[11] Mondal P. The puzzling chasm between cognitive representations and formal structures of linguistic meanings. Cogn Sci. 2022;46(9). DOI:10.1111/cogs.13200 36083283

[12] Shenefelt M, White H. If A, then B. New York: Columbia University Press; 2013.

[13] Partee BH (2004). Compositionality in formal semantics: selected papers by Barbara H. Partee. Oxford: Blackwell.

[14] Hampton JA, Winter Y, Eds. Compositionality and concepts in linguistics and psychology. Berlin: Springer; 2017.

[15] Pagin P. Compositionality, understanding, and proofs. Mind. 2009;118(471):713–737.

[16] Turney PD, Pantel P. From frequency to meaning: vector space models of semantics. J artif intell res. 2010;37:141–188.

[17] Rotaru AS, Vigliocco G, Frank SL. Modeling the structure and dynamics of semantic processing. Cogn Sci. 2018;42(8):2890–2917. 30294932 10.1111/cogs.12690 PMC6585957

[18] Fodor JA. The language of thought. Cambridge, Mass: Harvard University Press; 1975.

[19] Chierchia G, McConnell-Ginet S. Meaning in grammar. Cambridge, MA: MIT Press; 1990.

[20] Fodor JA. A theory of content and other essays. Cambridge, MA: MIT Press; 1990.

[21] Chomsky N. Language and thought. Wakefield: Moyer; 1993.

[22] Quilty-Dunn J, Porot N, Mandelbaum E. The best game in town: the re-emergence of the language of thought hypothesis across the cognitive sciences. Behavioral and Brain Sciences. 2022;1–55. DOI:10.1017/S0140525X22002849 36471543

[23] Kemmerer D, Gonzalez Castillo J, Talavage T, et al. Neuroanatomical distribution of five semantic components of verbs: evidence from fMRI. Brain Lang. 2008;107:16–43. 17977592 10.1016/j.bandl.2007.09.003

[24] Kemmerer D. Concepts in the brain: the view from cross-linguistic diversity. New York: Oxford University Press; 2019.

[25] Gibbs RW. Embodied experience and linguistic meaning. Brain Lang. 2003;84:1–15. 12537948 10.1016/s0093-934x(02)00517-5

[26] Crain S. The emergence of meaning. Cambridge: Cambridge University Press; 2012.

[27] Baggio G. Meaning in the brain. Cambridge, MA: MIT Press; 2018.

[28] Baggio G. Compositionality in a parallel architecture for language processing. Cogn Sci. 2021;45(5). DOI:10.1111/cogs.12949 34018238

[29] Arbib MA. Compositionality and beyond: embodied meaning in language and protolanguage. In: Werning M, Hinzen W Machery E, editors. The Oxford handbook of compositionality. New York: Oxford University Press; 2012. pp. 475–492.

[30] Krifka M. Some remarks on event structure, conceptual spaces and logical form. Theor Linguist. 2012;38:223–336.

[31] Gärdenfors P. Events and causal mappings modeled in conceptual spaces. Front Psychol. 2020;11:1078–1664. 32373016 10.3389/fpsyg.2020.00630 PMC7179668

[32] Zwarts J, Verkuyl H. An algebra of conceptual structure. Linguist Philos. 1994;17:1–28.

[33] Kitcher P. Explanatory unification. Philos Sci. 1981;48:507–531.

[34] Harner H, Khemlani S. Reasoning with *Want*. Cogn Sci. 2022;46(9). DOI:10.1111/cogs.13170 36007147

[35] Dale R, Duran ND. The cognitive dynamics of negated sentence verification. Cogn Sci. 2011;35(5):983–996. 21463359 10.1111/j.1551-6709.2010.01164.x

[36] Atmanspacher H. Contextual emergence of mental states. Cogn Process. 2015;16(4):359–364. 10.1007/s10339-015-0658-0 26018611

[37] Graben PB, Barrett A, Atmanspacher H. Stability criteria for the contextual emergence of macrostates in neural networks. Network Comput Neural Syst. 2009;20(3):178–196. 10.1080/09548980903161241 19731148

[38] Maimon A, Hemmo M. Does neuroplasticity support the hypothesis of multiple realizability? Philos Sci. 2022;89(1):107–127.

[39] Ambridge B, Pine JM, Rowland CF, et al. A semantics-based approach to the “no negative evidence” problem. Cogn Sci. 2009;33(7):1301–1316. 21585506 10.1111/j.1551-6709.2009.01055.x

[40] Goldberg A. Explain me this: creativity, competition, and the partial productivity of constructions. Princeton: Princeton University Press; 2019.

[41] Dobosz K, Duch W. Understanding neurodynamical systems via fuzzy symbolic dynamics. Neural Networks. 2010;23(4):487–496. 20045631 10.1016/j.neunet.2009.12.005

[42] Pelletier FJ. Compositionality and concepts—A perspective from formal Semantics and philosophy of language. In: Hampton J Winter Y, editors. Compositionality and concepts in linguistics and psychology. Berlin: Springer; 2017. pp. 31–94.

[43] Langacker R. Cognitive grammar: a basic introduction. New York: Oxford University Press; 2008.

[44] Lyu B, Choi HS, Marslen-Wilson WD, et al. Neural dynamics of semantic composition. Proc Nat Acad Sci. 2019;116(42):21318–21327. 31570590 10.1073/pnas.1903402116 PMC6800340

[45] Smirnova A, Lliev R (2014). Evidentiality in language and cognition: the view from construal level theory. In *Proceedings of the 36*^*th*^*Annual Meeting of the Cognitive Science Society*, Quebec City, 36, 2943–2948.

[46] Smirnova A. Evidentiality in abductive reasoning: experimental support for a modal analysis of evidentials. J Semant. 2021;38(4):531–570.

[47] Langacker R. Striving for control. In: Juana I-M-A, Carretero M, Hita JA, and van der Auwera J, editors. English modality. Berlin: Mouton de Gruyter; 2013. pp. 3–56.

[48] Chomsky N. The minimalist program. Cambridge, Mass: MIT Press; 1995.

